# Report of Two Cases of Abdominal Abscess

**Published:** 1884-03

**Authors:** W. H. Fitch

**Affiliations:** Rockford, Ill.


CHICAGO MEDICAL

JOURNAL & EXAMINER.

VOL. XLVIII.—MARCH, 1884.—NO. 3.

### Original Communications.

### ARTICLE I.

REPORT OF TWO CASES OF ABDOMINAL ABSCESS. By W. H. FITCH, M.D., Rockford, Ill.

Had I considered either of these cases simply perinephritic abscess, I should hardly have deemed them worthy of record, but they differed so essentially from cases of that kind that I have seen, that I have thought them of enough interest for brief detail.

CASE I.—Miss C., aged 21, consulted me first in October, 1877, for pityriasis capitis, tinea tarsi and eczema of external ear. She had scarlatina when six years old; this eruptive fever was followed by the above troubles and other constitutional dyscrasia, from which so many suffer for years and even a lifetime. Having prescribed the usual remedies at irregular intervals for some time with unsatisfactory results, in March, 1878, I made a somewhat more careful inquiry into the history of her case, and learned that in 1870 she was treated for ague for some time unsuccessfully by usual remedies, and obtained cessation of chills and fever by Indian cholagogue. This sickness was followed by extreme anæmia, which she ascribed to taking Fowler's Solution. However, she slowly regained her color, but suffered so much from a stinging pain in left side, headache, backache, and other neuralgic pains, that she was able to pursue her studies only at irregular intervals. The winter of 1873-74 she passed in California, and in the fall of 1875 went to Avon Springs for some months, but obtained no relief. While there she was taken with pain in the back, and fell on the floor; was carried to her room and was unable to walk or stand for a few days. Had several similar attacks after returning home, and was temporarily paraplegic. During the summer of 1877 she was severely ill with spinal spasmodic pains and neuralgia of sciatic nerve, and treated with blisters, hypodermic injections, liniments, etc., and after some weeks was again able to leave the house. She first consulted me concerning her back and side in March, 1878. Her appetite was capricious, sleep poor, menstruation painful, mind depressed, unable to take physical exercise except of gentlest kind.

A prominent homœopathic gynæcologist of Chicago had examined her pelvic organs on two days preceding her visit to me, and pronounced them healthy. Not wishing to prescribe on another's diagnosis, I made an examination, and found extensive erosion of the os uteri, endo-cervicitis, and anteflexion. The left hand on the abdomen detected a large tumefaction in the left abdominal region, extending from the crest of the ileum upward nearly to the ribs, and forward to within an inch and a half of the median line, that was somewhat tender to pressure, oval, circumscribed, non-fluctuating, and could not be reached per vaginam, thus excluding pelvic deposit or cellulitis.

This was quite a surprise to her, as she had been examined and treated by several physicians, and it had not been discovered—but was a solution, in part at least, of her years of suffering and despondency.

She was requested to paint the part with tr. iodine, and take alteratives and tonics, while the endo-cervicitis was treated with iodized phenol, borated tampons, etc., for a few weeks, with gradual improvement. In the beginning I had told her the swelling was uncommon, and would give her trouble. Not apparently satisfied with my prognosis or experience, unbeknown to me, she consulted another specialist in Chicago, who assured her it was quite common, and would disappear with the uterine trouble. Thus encouraged, she became his patient for three months, until the cervical catarrh was cured, but she was no better, and was recommended to the seaside for salt water baths, change of air, etc., also to be in charge of another specialist, where she remained until February, 1879, when she returned to me unimproved. An examination revealed very deep and somewhat indistinct fluctuation of the tumor. She was again put upon tonics, and flaxseed poultices applied until September, when aspiration was performed, and about one pint of pus withdrawn. By Dec. 19 the cavity had refilled, when I introduced the trocar a little above and halfway between the crest of the ileum and the median line, and withdrew 14 ounces of healthy pus, and washed the cavity out with a 5 per cent. sol. of carbolic acid. Jan. 21, 1880, the cavity had again partially refilled, and two ounces of pus were withdrawn, and again washed out with an 8 per cent. mixture of carbolic acid. April 16, slight deep fluctuation could again be detected, and six ounces of pus were withdrawn, and the cavity cleansed. After this there was no reaccumulation. It was necessary to introduce the trocar about two inches before the pyogenic capsule was reached, which was very tough, and penetrated with considerable difficulty.

The aspirations were not followed by any considerable pain or constitutional disturbance, and she was able to leave her bed and go down stairs on the same day. From the time of last aspiration her health and strength rapidly improved; the pain and lameness left the side, back, limbs, and head, and she has now enjoyed nearly four years of the best health of her life since a child.

CASE II.—April 12, 1882, was called to see Miss T., age 18, in full flesh, and every external appearance of perfect health. She gave me the following history: In April, 1880, while at college in the East, was suddenly seized one day with pain in the back, like lumbago, so she was scarcely able to move. Painted her back with iodine and applied various plasters, which relieved somewhat. In June the pain had shifted to the right side and was somewhat milder. In September she had pain in the back and both sides, being severer sometimes in one place then in another. During the fall and winter of 1881 she again had pain in back and sides, especially when sweeping, stooping or lifting. Had also considerable headache, especially behind the ears, increasing nervous irritability and chilly sensations, but no regular chills. In spring of 1881, pain in sides and back become constant and severe, and at times would run down right thigh and leg. Sleeping on right side increased the pain. In July, 1881, first consulted a physician, who used dry cups, liniments and blisters, but did not benefit. During the fall of 1881 she was conscious of growing weaker, having more constant pain and being less able to elevate right limb, go up or down stairs, get in or out of carriage, walk, dance, etc. A horse-back ride caused some pain, and on dismounting she was unable to step. Pain was somewhat in front of abdomen, and ran down to ankle. Could not sit long in one position. Appetite varying, sometimes loathing all kinds of food, at others craving sour articles. Was too nervous to sleep. In December went out of town for a month, as she was somewhat easier, and danced a little, which didn't cause much pain. Was pale at times, with dark rings under the eyes. At other times had flushed cheeks, and evidence of fever. Right leg and foot often felt cold and numb. Could not rest weight of body on right limb. Began to walk somewhat lame, and the right hip seemed higher than the other.

While away at this time, she slipped and fell on the pavement, which caused the return of so much pain that she was unable to stir for several days. After returning home from this month's absence, the right limb could not be moved except as she lifted it with her hands. Could not cough, laugh, or sneeze without increasing her pain. In February, 1882 the right leg seemed an inch longer than the other, and the whole side, arm and hand, were painful. Could not turn over in bed without sitting up. Had recently become fretful and emotional. Felt best, half reclining with hand pressing the side. Menstruation began at 14; always somewhat irregular, but painless, until the present trouble began two years ago. Her last physician said the "womb was tipped," but made no examination; the one before the last treated her for hysteria. Present condition: Pulse, 120; temperature, 101°. Patient well nourished and abundance of adipose tissue. Right half of abdomen most prominent, measuring an inch and half more than left. Also right thigh an inch larger than left, from œdema, which extends nearly to ankle. With the patient lying on her back a large deep fluctuating tumor was found in right side of abdomen, extending from near the liver to the crest of the ileum and a little below and more than half way to median line, sensitive to pressure. From my experience with Case 1, I at once made diagnosis of abdominal abscess, and advised aspiration. As this was the first time I had been called to the family, the parents wished a little delay unless the case was urgent. The patient was put on quinine, and mineral acids and poultices applied to side. May 1, Dr. J. saw the young lady in cousultation, but could not make out fluctuation, which was much obscured by a thick layer of adipose, and thought the case one of solid tumor of ovary. In this I could not concur. He, however, agreed to exploratory aspiration, which was done on May 3, the patient being under ether. The trocar was introduced three inches and three-quarters before the abscess cavity was penetrated, when sixteen ounces of laudable pus were withdrawn. It was introduced about one inch above crest of ileum and two inches toward median line.

The cavity was washed out with six per cent sol. carbolic acid. Operation not followed by reaction. May 29, cavity partially refilled and eight ounces aspirated; cavity washed out. July 10, fluctuation again detected, and six ounces of pus evacuated. Then about six ounces of an eight per cent sol. carbolic were injected through the canula into the cavity or some other place, as I was unable by any manipulation of my instruments to withdraw it, I withdrew the canula and reintroduced it with a trocar, but could not get back my solution, which represented about half an ounce of the pure acid. There was nothing to do but await results, which we did with utmost anxiety. When she recovered from the ether she complained of pain in region of abscess and a mixture of morphine and atropia was administered hypodermically, and repeated in the evening, when, as no other untoward symptom appeared, we felt the danger of poisoning was past. A mild grade of peritonitis followed, which was easily controlled, and on the 16th the tympanitis had subsided, the pulse and temperature were normal. From this date recovery was rapid, swelling of right side and limb disappeared, together with tilting of pelvis, lameness, fever, and pain. The cavity has not since refilled and the young lady is in perfect health. In my last communication with Case I, she said she had felt her trouble as a stinging pain for ten years; while Case II undoubtedly had its origin more than two years before I saw it. Neither one exhibited any tendency to pointing or spontaneous opening. Neither had urinary disturbances. If my carbolic acid sol. was only five per cent, there was still nearly $2\frac{1}{2}$ drams, but it was ordered eight per cent. Why it did not kill I am unable to answer. I have not space to give the differential diagnosis between these two cases and perinephritic abscess, only that the *tout ensemble* was unlike it.

